# Ferrous Glycinate (Fe-Gly) Supplementation Improves Growth Performance by Modulating Intestinal Immunity and Microbiota in Weaned Piglets

**DOI:** 10.3390/ani16030365

**Published:** 2026-01-23

**Authors:** Bijiang Li, Aimin Wu, Tingting Zhang, Ruiying Zhang, Huifen Wang, Quyuan Wang, Daiwen Chen

**Affiliations:** 1 Institute of Animal Nutrition, Sichuan Agricultural University, Chengdu 611130, China; 13458694168@163.com (B.L.); wuaimin0608@163.com (A.W.);; 2 Key Laboratory for Animal Disease-Resistance Nutrition of China Ministry of Education, Sichuan Agricultural University, Chengdu 611130, China

**Keywords:** weaned piglets, ferrous glycinate, iron metabolism, intestinal inflammation, gut microbiota

## Abstract

Iron is an essential nutrient for piglets, but iron sources vary greatly in bioavailability, and their effects on intestinal health remain unclear. In this study, 21-day-old weaned piglets were used to compare the effects of different iron sources (ferrous sulfate (FeSO_4_), ferric ammonium citrate (FAC), and ferrous glycinate (Fe-Gly)) on growth performance, intestinal inflammation, and gut microbiota. Compared to the FeSO_4_ group, the Fe-Gly group significantly increased the body weight of piglets at 35 days (*p* < 0.05), promoted the average daily feed intake (ADFI) and average daily gain (ADG) of piglets from day 21 to 35 (*p* < 0.01), and also markedly reduced the diarrhea rate of piglets (*p* < 0.01). Meanwhile, although FAC increased growth performance-related indicators (ADG, ADFI) in piglets, there was no significant statistical difference compared with FeSO_4_ (*p* > 0.10). Moreover, Fe-Gly supplementation significantly elevated serum iron levels and total iron-binding capacity (*p* < 0.01), while significantly reducing the iron content in colonic chyme (*p* < 0.0001). Both the Fe-Gly and FAC significantly improved the anti-inflammatory and antioxidant capacities of the piglets (*p* < 0.01). Interestingly, Fe-Gly primarily increased the abundance of *Lactobacillus*, thereby reducing the abundance of harmful bacteria such as *Escherichia coli*. Functional prediction using PICRUSt2 revealed that Fe-Gly supplementation tended to elevate the relative abundance of gut bacteria capable of carbohydrate metabolism and amino acid synthesis. In conclusion, this study demonstrated that dietary Fe-Gly supplementation improved systemic iron status, effectively reduce residual iron in the intestine, inhibit the proliferation of pathogenic bacteria in the gut, promote the growth performance and intestinal health of piglets, and reduce the diarrhea rate.

## Simple Summary

Iron is an essential nutrient for animals, playing a vital role in maintaining normal physiological functions. Thus, it is necessary to study how iron affects animal growth, development, and intestinal health, as well as the underlying mechanisms. In the swine industry, iron nutrition imbalance during the weaning period of piglets can impair intestinal health, lead to growth retardation, and severely reduce economic benefits. This study aimed to explore the effects and main mechanisms of different iron sources added to diets on the growth performance and intestinal health of weaned piglets. The results demonstrate that ferrous glycinate (Fe-Gly) not only effectively promotes the growth and intestinal epithelial development of piglets, but also reduces intestinal inflammation, enhances intestinal health, and lowers the risk of diarrhea. Additionally, due to its higher bioavailability, Fe-Gly leaves less residual iron in the intestines. This creates a more favorable environment for the growth of beneficial bacteria such as *Lactobacillus*, thereby further improving intestinal health. These findings deepen the understanding of the relationship between iron sources and intestinal health and provide a scientific basis for the application of efficient and healthy iron sources in the livestock industry.

## 1. Introduction

Nutrition is closely linked to animal health. As an essential nutrient, iron plays vital roles in physiological processes including DNA synthesis, oxygen transport, and energy metabolism, making it an essential trace element for piglets [1]. Both insufficient and excessive iron can severely affect the healthy development of piglets. Iron deficiency in piglets leads to iron-deficiency anemia, resulting in impaired intestinal barrier function and slow growth [2]. In contrast, iron overload induces the excessive production of free radicals, exacerbates systemic inflammation, and the excess iron in the intestines further promotes the growth of harmful bacteria, thereby compromising the health of the intestinal microbiota [3,4]. Therefore, how to balance the essentiality and toxicity of iron, as well as screen iron sources with high bioavailability that promotes intestinal health, has become a current research focus.

The long-standing practice of supplementing exogenous iron, particularly inorganic iron, in animal production faces challenges. Owing to its low bioavailability and susceptibility to antinutritional factors such as phytic acid [5], unabsorbed iron ions accumulate in the intestine, inducing intestinal oxidative stress. They also promote the proliferation of pathogenic microorganisms such as *E. coli* and *Salmonella*. This disrupts the structure of the intestinal microbiota and exacerbating intestinal inflammation through the activation of the TLR4/NF-κB signaling pathway [6]. This not only predisposes piglets to iron-deficiency anemia and impairs growth performance, but also increases fecal iron excretion, leading to environmental pollution and resource wastage [7,8]. In contrast, simple organic iron sources such as ferric ammonium citrate (FAC) offer advantages in reducing oxidative damage due to their superior chemical stability. However, studies indicate persistent limitations in their intestinal absorption efficiency and microbiota modulation [9]. Recently, amino acid-chelated iron (e.g., ferrous glycinate (Fe-Gly)) has garnered significant research interest due to its unique molecular structure. This small-molecule chelate can be directly absorbed through the intestinal amino acid transport system, achieving a bioavailability 30–50% higher than that of ferrous sulfate (FeSO_4_). Previous studies have demonstrated that Fe-Gly not only stably improves the daily weight gain of piglets [10], but also enhances barrier function by inhibiting pro-inflammatory cytokines secretion in the gut [11]. Differences in the chemical properties of various iron sources may affect piglet health through three primary pathways: (1) differential iron absorption and metabolism, influencing hematopoietic function and immune activity [12]; (2) varying amounts of unabsorbed iron remaining in the intestine, thereby altering the structure of the intestinal microbiota [13]; and (3) intrinsic differences in the chemical properties of the iron sources, directly affecting intestinal morphology development and antioxidant capacity [14]. However, current research focuses largely on evaluating the effects of single iron sources, and focused solely on the effects of iron sources on the growth performance and serum iron (SI) parameters of piglets, with a notable lack of systematic comparisons of multiple iron sources under the same experimental conditions. Particularly scarce are studies exploring the relationships among diarrhea control mechanisms, growth performance optimization, and differences in intestinal microbiota and metabolic products [15,16,17].

This study aims to compare the effects of three iron sources—FeSO_4_, FAC, and Fe-Gly—supplemented at 100 mg/kg on diarrhea rate, growth performance, iron nutritional status, and intestinal health indicators in weaned piglets to elucidate their differential mechanisms, thereby providing a theoretical basis for understanding the iron source-intestinal health relationship and scientific support for selecting efficient and environmentally friendly iron supplements to reduce antibiotic reliance.

## 2. Materials and Methods

### 2.1. Animal Ethics Statement

The experimental protocol was approved by the Animal Ethics and Welfare Committee of Sichuan Agricultural University (Approval No. 20240223).

### 2.2. Experimental Materials

FeSO_4_ was provided by the Ya’an Research Base of Sichuan Agricultural University (Ya’an, China), with an iron content of 20.1%. FAC and Fe-Gly were provided by Meishan Mengchuan Feed Co., Ltd. (Meishan, China), with iron contents of 16.5–18.5% and 17%, respectively. DLY weaned piglets were supplied by Meishan Wan Jiahao Xinhe Pig Farm (Meishan, China).

All reagents used in this study were obtained from commercial sources. Trizol reagent was purchased from Invitrogen (Carlsbad, CA, USA). ELISA kits for IL-1β, IL-6, IL-10, TNF-α, DAO, and ET were purchased from Enzyme-Linked Biotechnology (Yanchen, China). Serum malondialdehyde (MDA) and glutathione peroxidase (GPX) assay kits were purchased from the Jiancheng Bioengineering Institute (Nanjing, China). RNAiso Plus (Cat. #9109), gDNA Eraser, and TB Green^®^ Premix Ex Taq™ II (Cat. #RR820A) were purchased from Takara Bio (Dalian, China). Dulbecco’s Modified Eagle Medium (DMEM; Cat. #C11995500BT) was purchased from Gibco (Grand Island, NY, USA). Fetal bovine serum (Cat. #FSD500) was purchased from Excell (Suzhou, China).

### 2.3. Experimental Instruments

The following instruments were utilized in this study: a high-speed centrifuge (Eppendorf, Hamburg, Germany); a fluorescence microplate reader (Molecular Devices, San Jose, CA, USA); a low-temperature tissue grinder (Sichuan Century Shunhe Co., Ltd., Chendu, China); an electric thermostatic blast drying oven (Shanghai Fengling Laboratory Equipment Co., Ltd., Shanghai, China); a vortex mixer (Scientific Industries, Bohemia, NY, USA); an automatic blood analyzer (Hitachi, Japan); and a NanoDrop spectrophotometer (Thermo Fisher Scientific, Grand Island, NY, USA). Reverse transcription was performed using the RT Master Mix (Takara Bio, Dalian, China; Cat. #RR036A), and quantitative real-time PCR was conducted on a QuantStudio 6 Real-Time PCR System (Thermo Fisher Scientific, Grand Island, NY, USA).

### 2.4. Experimental Design

The study adopted a completely randomized design. A total of 72 healthy 21-day-old DLY (Duroc × Landrace × Yorkshire) weaned barrows with an initial body weight of 7.06 ± 0.23 kg were randomly divided into three dietary treatments. Each treatment had six replicates with four piglets per replicate. The randomization was performed using a random number table method to ensure that each experimental unit had an equal probability of being assigned to any treatment group, and there was no blocking factors involved in the design. Different iron sources were added to the basal diet (without exogenous iron supplementation), namely the FeSO_4_ group, FAC group, and Fe-Gly group. Iron was supplemented at a dose of 100 mg/kg of the diet. This dosage was established by preceding experiments from our research team. The experiment lasted for 35 days. The pen was considered the experimental unit for all analyses of growth performance, fecal microbiota, and diarrhea rate, as the dietary treatments were applied at the pen level.

### 2.5. Experimental Diet

The diet used in this trial was formulated in accordance with the NRC (2012) [18] nutritional requirements for 5–11 kg piglets (Table 1).

### 2.6. Sample Collection

Blood sampling: One pig with average body weight and good health status was selected from each replicate (*n* = 6) and fasted for 12 h to blood collection. A 15 mL of blood was drawn from the anterior vena cava of each pig. From this sample, 2 mL was transferred into an EDTA-coated tube for hematological analysis. The remaining blood was left at room temperature for 30 min, then centrifuged at 3000 r/min for 15 min to prepare serum, which was stored at −20 °C for subsequent biochemical assays.

Fecal sampling: A sterile cotton swab was dipped in a small amount of sterile physiological saline (moistened appropriately, avoiding excessive dilution of the sample) and gently inserted 2–3 cm into the rectum of the piglet. The swab was rotated gently 1–2 times to allow the swab head to adsorb an adequate amount of fresh feces, then slowly withdrawn. After sampling, the centrifuge tube cap was immediately tightened, and the outer wall of the tube was wiped with 75% alcohol to remove potential environmental contaminants. After collection, equal amounts of fecal samples from each piglet in a pen were combined (pooled) into one sterile tube as a composite sample, which was then held at 4 °C prior to processing.

### 2.7. Production Performance and Diarrhea Severity

On days 0, 14, 21, and 28 of the trial, piglets were fasted overnight and individually weighed. The average daily gain (ADG), average daily feed intake (ADFI), feed-to-gain ratio (F/G) were calculated for each pen.

ADG = (final weight − initial weight)/number of days

ADFI = total feed intake/number of days

F/G = ADG/ADFI

Diarrhea scores were visually assessed three times a day as previous described [19].

Diarrhea index = the sum of diarrhea scores/(numbers of piglets per pen × experimental days × assessed times per day).

Diarrhea scoring: 0 = normal, solid feces; 1 = mild diarrhea, soft and loose feces; 2 = moderate diarrhea, soft and unformed feces; 3 = severe diarrhea, watery feces.

Diarrhea incidence was calculated based on the daily scoring results. When the fecal score of an individual piglet was ≥2 on a given day, it was recorded as a diarrheal event. The diarrhea rate for each replicate pen was calculated using the following formula:

Diarrhea incidence (%) = [total number of diarrhea incidents/(total number of piglets × experimental days)] × 100

### 2.8. Serum Inflammation and Antioxidant Indicators

Serum levels of tumor necrosis factor-α (TNF-α), interleukin-1β (IL-1β), interleukin-6 (IL-6), and interleukin-10 (IL-10), as well as the total antioxidant capacity (T-AOC) and malondialdehyde (MDA) content in serum, were measured using enzyme-linked immunosorbent assay (ELISA) kits and biochemical assay kits, respectively, in accordance with the manufacturer’s protocols.

### 2.9. Serum Iron Indicators

Total iron-binding capacity (TIBC), unsaturated iron-binding capacity (UIBC), SI, and transferrin saturation (TF) were measured using kits (Iron Standard Reagent, Pointe Scientific, Canton, MI, USA). Among these, TIBC = SI + UIBC, and TF = SI/TIBC × 100%

### 2.10. Fecal Iron Content Measurement

Approximately 50 mg of feces was added to 10 mL of ultrapure-grade nitric acid and digested using a microwave digestion system. The digestate was then heated at 150–200 °C to evaporate the acid. When the volume was reduced to 0.5 mL, it was diluted to 10 mL with 2% ultrapure-grade dilute nitric acid and filtered through a 0.45 μm aqueous filter membrane. The filtrate was collected and further diluted 10-fold before iron content was measured using Inductively Coupled Plasma Mass Spectrometry (ICP-MS). The fecal iron content was determined on an as-is basis.

### 2.11. Cell Culture

The porcine intestinal epithelial cell line (IPEC-J2) was obtained from the American Type Culture Collection (ATCC) (Manassas, VA, USA) and cultured in DMEM supplemented with 10% (*v*/*v*) fetal bovine serum, 100 U/mL penicillin, and 100 U/mL streptomycin. The cultures were maintained at 37 °C in a 5% CO_2_ atmosphere with water-saturated humidity.

### 2.12. Cell Viability Assay

When IPEC-J2 cells reached 70–80% confluence in 96-well plates, the original culture medium was removed. Then, 90 μL of fresh complete medium and 10 μL of different concentrations of FeSO_4_, FAC, or Fe-Gly were added to each well. Each concentration was tested in six replicate wells. The plates were incubated under standard culture conditions (37 °C, 5% CO_2_).

At 24 h after treatment, the medium in each well was replaced with 100 μL of fresh complete medium, followed by the addition of 10 μL of CCK-8 reagent per well. After further incubation for 4 h under the same conditions, the absorbance was measured at 450 nm using a microplate reader.

Cell viability was calculated using the following formula:

Cell viability (%) = [(OD_treatment_ − OD_blank_)/(OD_control_ − OD_blank_)] × 100%

### 2.13. Real-Time Quantitative PCR

Total RNA of cells was extracted using RNAiso Reagent and the RNA samples were reversely transcribed into cDNA using PrimeScript RT reagent kit with gDNA Eraser according to manufacturer’s instructions. The concentration and purity of total RNA isolation were determined before reverse transcription PCR by DU 640 UV spectrophotometer detection. RT-qPCR was performed using the QuantStudio 6 Real-Time PCR System and the SYBR Green PCR Master Mix. Each reaction (10 μL) contained 5 μL TB Green Premix, 0.4 μL forward primer (10 μM), 0.4 μL reverse primer (10 μM), 2 μL cDNA template, and 2.2 μL nuclease-free water. The cycling protocol was as follows: initial denaturation at 95 °C for 30 s, followed by 40 cycles of 95 °C for 5 s and 60 °C for 30 s. The primers used were listed in Table 2. β-actin was used as an internal control to normalize the expression of the selected genes and the relative expression levels of these target genes were calculated by using the 2^−∆∆Ct^ method. All primers were designed, when possible, to span exon–exon junctions and were synthesized by Sangon Biotech (Shanghai, China).

### 2.14. 16S rRNA Sequencing

The DNA of colonic content was extracted by E.Z.N.A.^®^ Stool DNA Kit. For 16S rRNA sequencing, the DNA samples were sent to the Novogene Company (Beijing, China). Gut microbiota was analyzed based on the 16S rRNA gene V3–V4 sequencing of DNA samples. The V3–V4 region of the 16S rRNA gene was amplified using universal primers, including the forward primer 341F (5′-CCTAYGGGRBGCASCAG-3′) and reverse primer 806R (5′-GGACTACNNGGGTATCTAAT-3′). PCR reactions were performed using 15 μL of Phusion^®^ High-Fidelity PCR Master Mix (New England Biolabs, Ipswich, MA, USA), 2 μM of forward and reverse primers, and approximately 10 ng of template DNA. The thermal cycling protocol consisted of an initial denaturation step at 98 °C for 1 min, followed by 30 cycles of denaturation at 98 °C for 10 s, annealing at 50 °C for 30 s, and elongation at 72 °C for 30 s. A final extension step was performed at 72 °C for 5 min. The quantification and qualification of PCR products were performed by electrophoresis on 2% agarose gels. Sequencing libraries were generated using the TruSeq^®^ DNA PCR-Free Sample Preparation Kit (Illumina, San Diego, CA, USA), and the library was sequenced on an Illumina NovaSeq platform (Illumina). The Quantitative Insights into Microbial Ecology2 (QIIME2) software (Version QIIME2202202) was used to carry out the Bioinformatics analysis of sequencing data. Microbial function analysis was conducted using the phylogenetic investigation of communities by reconstruction of unobserved states (PICRUST2) based on 16S rRNA gene amplicon sequences. The functional annotation of PICRUST2 prediction was obtained based on several gene family databases such as Kyoto Encyclopedia of Genes and Genomes11 (KEGG) orthologs (KOs) and Enzyme Commission numbers [20,21].

### 2.15. Statistical Analysis

Statistical analysis was performed using Excel 2021 and GraphPad Prism 9.5.1. Data are presented as mean ± SEM. One-way ANOVA followed by Tukey’s multiple comparisons test was conducted. A significance level of 0.05 < *p* < 0.1 was considered a significant trend, *p* < 0.05 was considered statistically significant, and *p* < 0.01 was considered highly statistically significant.

## 3. Results

### 3.1. Effects of Different Dietary Iron Sources on Growth Performance and Diarrhea Rate in Piglets

As shown in Table 3, at day 35 of the trial, the body weight of the piglets in the Fe-Gly group was significantly higher than that of the FeSO_4_ group (*p* < 0.05). Over the entire 35-day trial, Fe-Gly significantly increased ADG compared to FeSO_4_ (*p* < 0.05). During the period of day 21 to 35, piglets in the Fe-Gly group also exhibited improved ADG (*p* < 0.01) and ADFI (*p* < 0.05). It is noteworthy that during the period of day 1 to 14, the diarrhea incidence in the Fe-Gly group was highly significantly lower than that in the FeSO_4_ group (*p* < 0.01). No significant differences were observed in various growth performance parameters between the FAC group and the FeSO_4_ group, but the diarrhea rate in the FAC group was significantly lower than that in the FeSO_4_ group (*p* < 0.05).

### 3.2. Effects of Different Dietary Iron Sources on Serum Iron and Fecal Iron in Piglets

**Figure 1:**
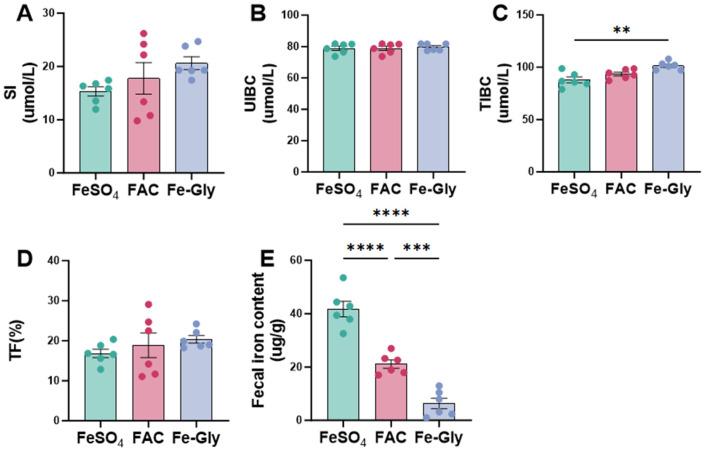
Effects of different iron sources on serum iron and fecal iron in weaned piglets. (**A**) Serum iron (SI). (**B**) Unsaturated iron-binding capacity (UIBC). (**C**) Total iron-binding capacity (TIBC). (**D**) Transferrin saturation (TF). (**E**) Fecal iron content. Statistical significance is indicated as follows: ** *p* < 0.01; *** *p* < 0.001; **** *p* < 0.0001.

As shown in Figure 1, no significant differences were observed in SI among the three groups; however, compared with the FeSO_4_ group, Fe-Gly showed a 35% numerical increase in SI (*p* = 0.15; Figure 1A). Similarly, no significant differences were observed in UIBC among the three groups (*p* > 0.10; Figure 1B), indicating that the iron sources primarily influenced the metabolism or transport of bound iron rather than the potential binding capacity of serum. Accordingly, the TIBC in the Fe-Gly group was significantly higher than in the FeSO_4_ group (*p* < 0.01; Figure 1C). This enhanced TIBC indicates that Fe-Gly more effectively promotes the iron-binding capacity of serum transferrin protein. Consistently, transferrin levels were elevated in the Fe-Gly group compared to the FeSO_4_ group, with a similar trend observed versus the FAC group (*p* > 0.10; Figure 1D). These findings collectively demonstrate that the iron source modulates the transferrin-mediated iron transport pathway, which aligns with the observed changes in SI.

Fecal iron content in the FeSO_4_ group was extremely significantly higher than that in the FAC and Fe-Gly groups (*p* < 0.001, *p* < 0.0001). Furthermore, the Fe-Gly group exhibited significantly lower fecal iron content than FAC group (*p* < 0.001; Figure 1E). These results suggest that the intestinal absorption efficiency of Fe-Gly and FAC was superior to that of FeSO_4_, with Fe-Gly having the highest absorption efficiency, as reflected by the lowest level of fecal iron excretion.

### 3.3. Regulatory Effects of Different Iron Sources on Inflammatory Response and Antioxidant Homeostasis in Weaned Piglets

**Figure 2:**
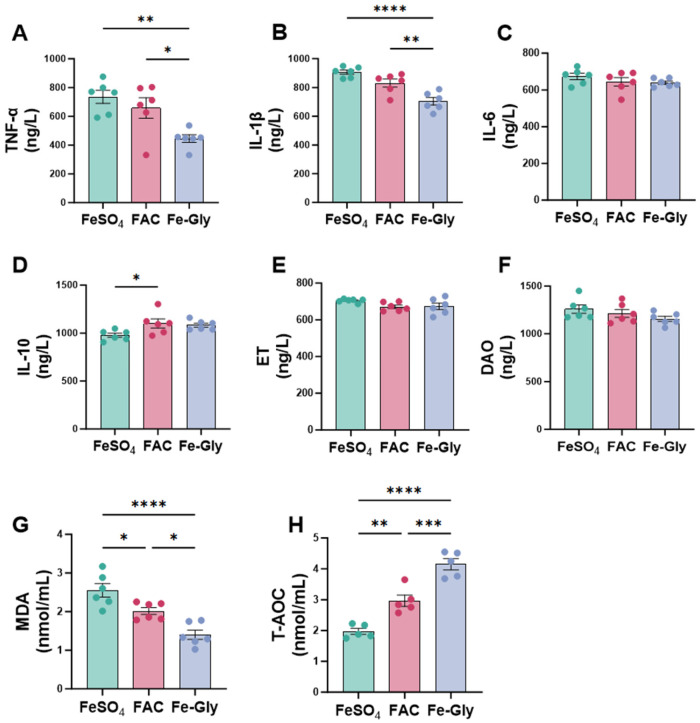
Effects of different iron sources on serum inflammatory markers and antioxidant capacity in weaned piglets. (**A**) Tumor necrosis factor-α (TNF-α). (**B**) Interleukin-1β (IL-1β). (**C**) Interleukin-6 (IL-6). (**D**) Interleukin-10 (IL-10). (**E**) Endothelin (ET). (**F**) Diamine Oxidase (DAO). (**G**) Malondialdehyde (MDA). (**H**) Total Antioxidant Capacity (T-AOC). Statistical significance is indicated as follows: * *p* < 0.05; ** *p* < 0.01; *** *p* < 0.001; **** *p* < 0.0001.

As shown in Figure 2, different iron sources significantly modulated serum inflammatory cytokines, intestinal barrier function, and antioxidant capacity in weaned piglets. Regarding pro-inflammatory cytokines, the serum TNF-α and IL-1β in Fe-Gly group were highly significantly lower than that in the FeSO_4_ group (*p* < 0.01 and *p* < 0.0001, respectively; Figure 2A,B). IL-1 in the Fe-Gly group was also lower than in the FAC group (*p* < 0.01). In contrast, IL-6 levels did not differ among groups (Figure 2C). For anti-inflammatory cytokine IL-10, levels in the FAC group were also significantly higher than that in the FeSO_4_ group (*p* < 0.05), while no significant difference was found between the Fe-Gly and FAC groups (Figure 2D). This indicates that both FAC and Fe-Gly supplementation promoted anti-inflammatory responses compared to FeSO_4_, with Fe-Gly demonstrating a more pronounced effect.

For systemic indicators of intestinal barrier function, no significant differences were observed in serum endotoxin (ET) and diamine oxidase (DAO) levels among the three groups (Figure 2E,F), suggesting that the iron sources had minimal impact on systemic indicators of intestinal barrier damage under the experimental conditions. In contrast, the serum MDA content was significantly higher in the FeSO_4_ group compared to the FAC and Fe-Gly groups (FeSO_4_ vs. FAC, *p* < 0.05; FeSO_4_ vs. Fe-Gly, *p* < 0.0001). Moreover, the MDA level in the Fe-Gly group was significantly lower than that in the FAC group (*p* < 0.05). The T-AOC was significantly higher in the Fe-Gly group than in the FeSO_4_ and FAC groups (Fe-Gly vs. FeSO_4_, *p* < 0.0001; Fe-Gly vs. FAC, *p* < 0.001). The FAC group also exhibited higher T-AOC than the FeSO_4_ group (*p* < 0.01), indicating that Fe-Gly more effectively improved the body’s antioxidant status (Figure 2G,H).

### 3.4. Effects of Different Iron Sources on the Development and Barrier Function of Intestinal Epithelial Cells

**Figure 3:**
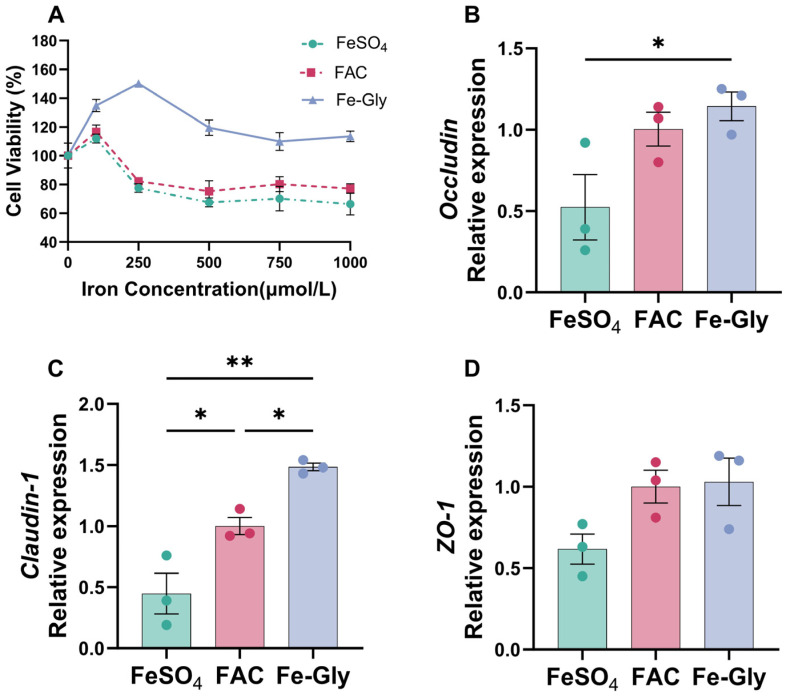
Effects of different iron sources on the proliferation and barrier function of intestinal epithelial cells. (**A**) Cell Viability. (**B**) *Occludin* mRNA. (**C**) *Claudin-1* mRNA. (**D**) zonula occludens-1 (*ZO-1*) mRNA. Data are expressed as mean ± SEM for 3 cell culture replicates in each group. Statistical significance is indicated as follows: * *p* < 0.05; ** *p* < 0.01.

To further elucidate the direct regulatory effects of different iron sources on the development and function of intestinal epithelial cells, we utilized IPEC-J2 cells as a model to analyze the impact of various iron sources on cell viability and the expression of intestinal barrier-related proteins. First, we used the CCK-8 kit to assess the effects of different iron sources at various concentrations (0, 100, 250, 500, 1000 μmol/L) on cell viability after 24 h of incubation. The results showed that Fe-Gly significantly promoted cell viability at medium concentrations (0–500 μmol/L) (*p* < 0.05), while high concentrations showed no significant toxicity to the cells. In contrast, FeSO_4_ and FAC significantly inhibited cell proliferation at concentrations exceeding 100 μmol/L (*p* < 0.05; Figure 3A). Subsequently, based on the cell viability results, all iron sources were applied at a concentration of 100 μmol/L to treat the cells. After 24 h of incubation, the expression of intestinal barrier-related proteins was measured using RT-qPCR. The results demonstrated that, compared to the FeSO_4_ group, the Fe-Gly group significantly upregulated the expression of *Occludin* and *Claudin-1* (*p* < 0.05, *p* < 0.01; Figure 3B,C). No significant differences were observed in ZO-1 expression among the three groups, but a trend towards increase was noted in the other two groups compared to the FeSO_4_ group (0.05 < *p* < 0.10; Figure 3D).

### 3.5. Effects of Different Iron Sources on Gut Microbiota Diversity and Composition in Piglets

**Figure 4:**
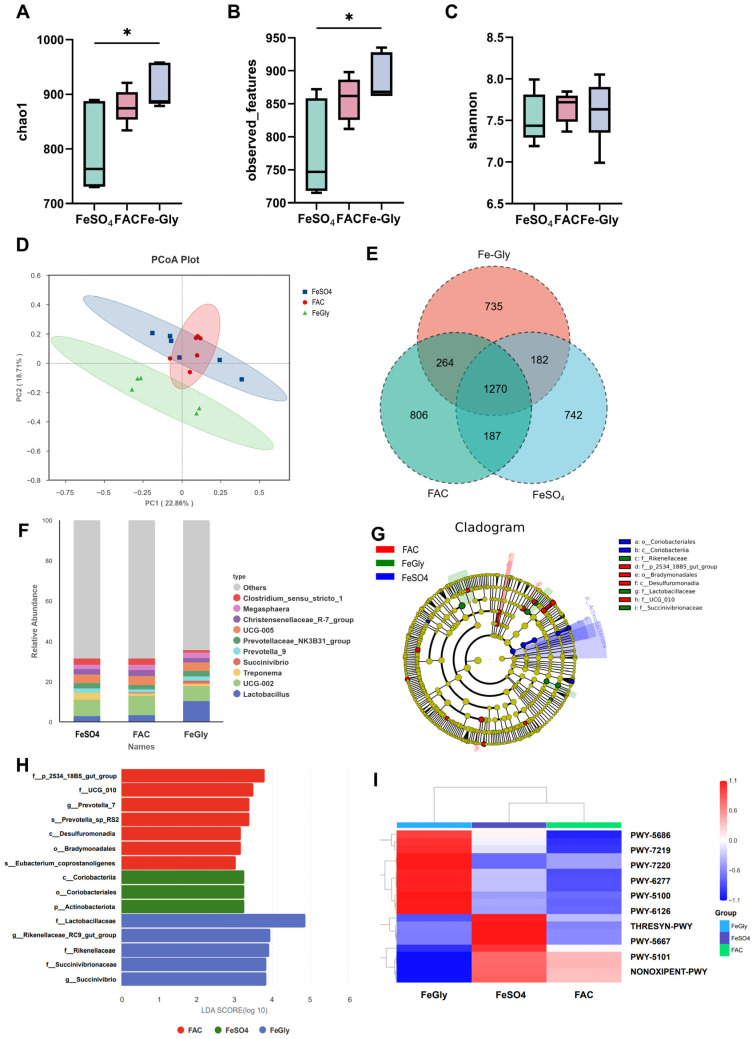
Effects of different iron sources on the diversity and composition of gut microbiota in weaned piglets. (**A**) Chao index. (**B**) Observed—features index. (**C**) Shannon index. (**D**) PcoA analysis. (**E**) Venn diagram data. (**F**) Community abundance analysis at the genus level. (**G**) GCircos plots analysis at the genus level. (**H**) LDA discriminant histogram. (**I**) PICRUSt2 functional prediction. Statistical significance is indicated as follows: * *p* < 0.05.

Given the crucial role of iron in shaping gut microbiota, while is closely linked to piglet health, growth, and development, we employed 16S rRNA sequencing to observe changes in the colonic microbiota of piglets receiving different iron sources. Alpha diversity analysis, showed that the Fe-Gly group significantly increased microbial diversity, as evidenced by significantly higher Chao1 index (richness), observed OTUs (richness), and Shannon index (diversity and evenness) compared to the FeSO_4_ and/or FAC groups (Figure 4A–C). Consistent with this, the principal coordinates analysis (PCoA) of beta diversity showed a clear separation of the microbial composition in the Fe-Gly group from the other two groups (Figure 4D).

Venn diagram analysis identified unique and shared microbial taxa among the treatment groups (Figure 4E). Further compositional analysis revealed that Fe-Gly supplementation significantly increased the relative abundance of *Lactobacillus* at the genus level compared to the FeSO_4_ and FAC groups (Figure 4F). LEfSe analysis (LDA score > 3) identified distinct microbial biomarkers for each group (Figure 4G,H). The FeSO_4_ group was characterized by an enrichment of *Actinobacteriota* and *Coriobacteriales*, taxa often associated with intestinal inflammation and iron metabolism dysbiosis. The FAC group showed enrichment of UCG_010, a genus linked to intestinal mucosal immunity and short-chain fatty acid (SCFA) synthesis. In contrast, the Fe-Gly group was markedly enriched with beneficial families including *Lactobacillaceae* and *Succinivibrionaceae*, which are known to regulate intestinal pH, carbohydrate metabolism, and SCFA production. Functional prediction of microbial communities using PICRUSt2 indicated that pathways enriched in the Fe-Gly group were primarily related to carbohydrate metabolism and amino acid synthesis, suggesting that microbial changes were closely associated with nutrient breakdown and energy production (Figure 4I).

### 3.6. Spearman’s Correlation Analysis

**Figure 5:**
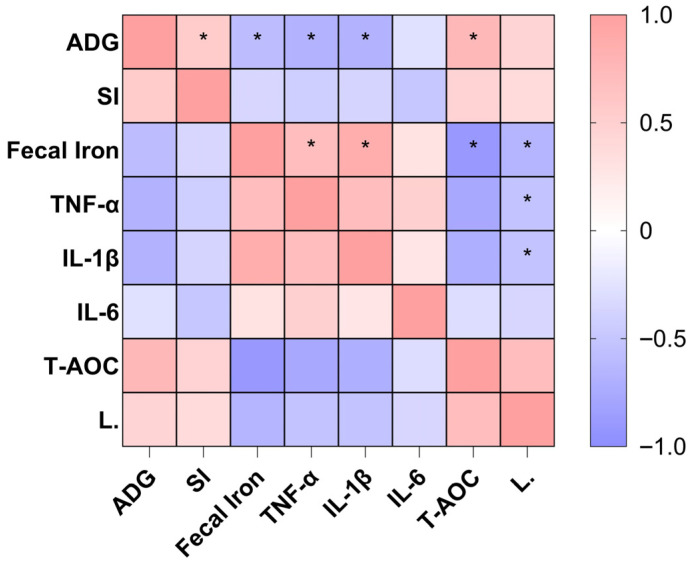
Chyme iron correlates with growth performance, inflammation, and microbiota of piglets. *L.*, *Lactobacillus.* Red indicates a positive correlation, and blue indicates a negative correlation. Statistical significance is indicated as follows: * *p* < 0.05.

From the correlation analysis in Figure 5, it can be seen that the Fecal Iron content is significantly and strongly positively correlated with proinflammatory cytokines (TNF-α, IL-1β) (*p* < 0.05), while it is significantly negatively correlated with T-AOC, ADG, and L. (*Lactobacillus*) (*p* < 0.05). This indicates that excessive iron accumulation in the intestines can trigger intestinal inflammation and oxidative stress responses in piglets, disrupt the intestinal flora homeostasis, ultimately leading to impaired growth performance.

## 4. Discussion

During the weaning period, piglets face multiple stressors—including separation from sows, transition to group living, and shifting from suckling milk to solid feed—that challenge their intestinal health [22]. Iron, as an essential trace element for animals, participates in various physiological processes and plays a critical role in maintaining intestinal health in piglets. Supporting this, Chen et al. (2008) [23] reported that organic iron sources, notably Fe-Gly, significantly improved piglet body weight and daily weight gain in weaned piglets. Our results are consistent with these findings, confirming that organic chelated iron, particularly Fe-Gly, possesses high bioavailability and is more efficiently absorbed in the piglet intestine. This ensures sufficient iron availability for growth, thereby promoting efficient utilization of nutrients. The significantly lower incidence of diarrhea in the Fe-Gly group compared to the FeSO_4_ group aligns with the findings of Zhang et al. (2000) [24] who also found that organic iron sources reduced diarrhea rates in piglets. Additionally, the high bioavailability of Fe-Gly not only benefits animal health but also reduces iron excretion, thereby lessening environmental pollution [25].

The chemical form of iron is a key factor determining iron absorption and metabolic efficiency. FeSO_4_, as an inorganic iron source, has low solubility and bioavailability, resulting in poor intestinal absorption efficiency. Consequently, a substantial amount of iron is excreted in feces, leading to the highest fecal iron content in the FeSO_4_ group and limiting improvements in indicators such as SI and TF. FAC, as an organic chelated iron, demonstrates superior solubility and intestinal absorption efficiency compared to inorganic FeSO_4_, effectively enhancing SI levels and transferrin expression while reducing fecal iron residue. However, the results from Shi et al.(2023) [17] showed that the SI content was lower with dietary Fe-Gly supplementation compared to ferrous sulfate, a discrepancy that may be attributed to inconsistent dosing among the three iron sources in their study. Huang et al. (2023) [26] found that organic iron-peptide chelates form soluble complexes with iron, preventing iron precipitation. These complexes are absorbed via intestinal peptide transport pathways and regulate enterocyte proliferation and differentiation through interactions with intestinal iron absorption mechanisms [5]. This explains why Fe-Gly in this study was more readily absorbed in the intestine, resulting in significantly lower fecal iron residue compared to FeSO_4_ and FAC. Moreover, efficiently absorbed iron enhances the iron-binding capacity of transferrin in serum, as evidenced by the significantly elevated TIBC. The lack of significant differences in UIBC among the three groups may be attributed to the fact that different iron sources primarily influence iron absorption and transport processes (e.g., metabolism of bound iron) rather than the total capacity of iron-binding proteins. In summary, both Fe-Gly and FAC were more effective than FeSO_4_ at enhancing iron absorption and SI metabolism in weaned piglets. Notably, Fe-Gly likely possesses the highest intestinal absorption efficiency, minimizing intestinal iron residue and optimizing SI transport potential.

As an inorganic iron source, FeSO_4_ is prone to triggering the Fenton reaction due to free iron overload [27], generating excessive reactive oxygen species (ROS) and activating inflammatory signaling pathways such as NF-κB. This leads to the overproduction of pro-inflammatory cytokines like TNF-α and IL-1β [28,29]. In contrast, FAC and Fe-Gly, owing to their higher bioavailability, minimize free iron-induced oxidative stress. Consequently, they downregulate pro-inflammatory cytokines and upregulate anti-inflammatory cytokines (e.g., IL-10). Specifically, Fe-Gly is absorbed via amino acid transport systems, providing functional iron more precisely to immune cells while avoiding the side effects of iron overload. This makes it particularly effective in regulating anti-inflammatory balance [30]. MDA, a marker of lipid peroxidation, is positively correlated with the degree of oxidative damage. T-AOC reflects the comprehensive capacity of antioxidant enzymes (e.g., SOD, CAT) and non-enzymatic antioxidants (e.g., GSH) [26]. The lowest MDA and highest T-AOC levels in the Fe-Gly group are consistent with previous studies [31]. This may be attributed to two factors: first, the chelated form of iron reduces oxidative stress induced by free iron; second, glycine, as a precursor of antioxidants like glutathione, synergistically enhances the activity of the antioxidant system when chelated with iron, significantly improving T-AOC [32,33]. In comparison, although FAC is an organic iron source, it lacks the synergistic antioxidant potential of amino acids, resulting in a weaker T-AOC improvement effect than Fe-Gly.

Intestinal development and barrier function are critical components for ensuring the healthy growth of piglets. To elucidate the comparative advantages of Fe-Gly at the cellular level, we conducted in vitro cell experiments, which confirmed that Fe-Gly significantly promotes the proliferation and growth of intestinal epithelial cells. In contrast, excessive FeSO_4_ and FAC exhibited marked inhibitory effects on cell proliferation. This finding further corroborates that the low absorption rate of FeSO_4_ readily leads to substantial iron accumulation in the intestine, thereby compromising intestinal health. Furthermore, Li et al. (2025) [34] found that dietary supplementation with Fe-Gly can significantly enhance the intestinal barrier function of piglets. This study further corroborates that Fe-Gly significantly upregulates the expression of tight junction proteins, directly strengthening the physical barrier function of the intestinal epithelium. The underlying mechanism may lie in the unique stable chelated structure of Fe-Gly and its potential to mimic peptide transport pathways. This pathway helps reduce oxidative stress triggered by free iron, thereby maintaining the integrity of tight junction structures and strengthening barrier function. However, as no significant differences were observed in serum DAO and ET levels, the improvement in intestinal barrier function appears to be localized at the cellular level rather than a systemic physiological change. These cellular-level findings provide key molecular mechanistic support for the observed phenotypic outcomes of improved growth performance and reduced diarrhea rate in the Fe-Gly group in animal trials.

The gut microbiota plays a crucial role in nutrient metabolism and health maintenance in piglets [35,36]. In this study, Fe-Gly features a unique amino acid-chelated structure that facilitates efficient intestinal absorption. This enhanced absorption minimizes the adverse effects of free iron on the gut microbiota, fostering a favorable environment for a more diverse microbial community, particularly beneficial bacteria, and thereby enhancing both alpha and beta diversity of the gut microbiota. McMillen et al. (2022) [37] found that different iron levels significantly influence the gut microbiota. Certain bacteria, such as *Lactobacillus*, do not require iron, which provides them with a competitive advantage in low-iron environments, while others, such as *E. coli*, gain an ecological niche under high-iron conditions. This finding aligns with the results of the present study. Concurrently, research indicates that *Lactobacillus* helps lower intestinal pH through lactate production, thereby inhibiting the colonization of harmful bacteria and reducing the incidence of diarrhea [38]. Furthermore, *Succinivibrionaceae* participates in carbohydrate and short-chain fatty acid metabolism, providing more energy and nutrients for piglet growth [39].

In contrast, FeSO_4_ has low intestinal absorption efficiency. The residual iron exerts selective pressure on microorganisms, leading to the enrichment of taxa such as Coriobacteriales, which tend to proliferate abnormally under iron metabolism imbalance [40]. It also increases the abundance of potentially harmful bacteria like Actinobacteriota, raising the risk of intestinal inflammation and diarrhea [41]. Although FAC is an organic chelated iron, its effects on promoting beneficial bacteria growth and enhancing microbial richness are less pronounced than those of Fe-Gly. Functional prediction via PICRUSt2, followed by KEGG pathway enrichment analysis, revealed that Fe-Gly most effectively modulates the metabolic pathways of gut microbiota enabling more efficient participation in nutrient breakdown and energy production, thereby indirectly promoting the growth and health of weaned piglets. However, it should be noted that these findings are based on predicted metagenomic data and require further validation through metabolomic studies. Collectively, these findings indicate that alterations in gut microbiota may be a critical factor underlying the differential effects of various iron sources on piglet growth performance and intestinal health.

## 5. Conclusions

Based on the results, dietary supplementation with Fe-Gly significantly increased the average daily gain of piglets by approximately 24% and increased average daily feed intake by about 15%, while reducing the diarrhea incidence by around 40% compared to the FeSO_4_ group. Furthermore, Fe-Gly significantly enhanced serum TIBC and decreased fecal iron content, indicating reduced intestinal iron accumulation. The concurrent reduction in serum pro-inflammatory cytokines and oxidative stress markers, along with an increased abundance of Lactobacillus in feces, collectively support the interpretation that Fe-Gly may alleviate intestinal inflammatory status and promote a healthier gut microenvironment, which likely contributed to the improvement in growth performance.

## Figures and Tables

**Table 1:** Composition of basal diet (as-fed basis).

Ingredients	Levels (%)	Nutrient Levels ^2^	Levels
Corn (CP, 7.8%) ^1^	45.73	DE (Mcal/kg)	3.30
Extruded corn (CP, 7.9%)	18.00	CP (%)	17.97
Soybean meal (CP, 46%)	6.00	CF (%)	2.12
Extruded Soybean	7.50	Total phosphorus (%)	0.62
Soybean protein concentrate (CP, 65%)	7.00	Calcium (%)	0.72
Low protein whey powder (CP, 3%)	5.00	SID ^3^ Lys (%)	1.35
Soybean oil	0.83	SID-Met (%)	0.50
Fish meal (CP, 67%)	4.00	SID-Thr (%)	0.79
Limestone	0.54	SID-Trp (%)	0.22
Dicalcium phosphate	0.88	SID-Met + Cys (%)	0.74
L-Lys·HCl	0.58		
DL-Met	0.11		
Thr	0.24		
Trp	0.06		
NaCl	0.40		
Sucrose	1.75		
Lactic acid	1.00		
Multi-enzyme Preparation	0.10		
Mineral premix ^4^	0.20		
Vitamin premix ^5^	0.05		
Mold Inhibitor	0.01		
Sweetener	0.02		
Total	100.00		

^1^ (CP, X%): The values represent the guaranteed analysis of the ingredients used in this experiment. ^2^ Nutrient levels were calculated levels. ^3^ SID: standardized ileal dgestibility. ^4^ Mineral Premix Composition (per kg of complete diet): Zinc (from ZnSO_4_·H_2_O): 100 mg; Copper (from CuSO_4_·5H_2_O): 6 mg; Manganese (from MnSO_4_·H_2_O): 4 mg; Selenium (from Na_2_SeO_3_): 0.3 mg; Iodine (from KI): 0.25 mg. ^5^ Vitamin Premix Composition (per kg of complete diet): Vitamin A: 15,000 IU; Vitamin D3: 5000 IU; Vitamin E: 40 IU; Vitamin K3: 5 mg; Biotin: 2.5 mg; Folic acid: 2.5 mg; Niacin: 50 mg; Pantothenic acid: 25 mg; Vitamin B2: 12.5 mg; Vitamin B6: 6 mg; Vitamin B1: 5.0 mg; Vitamin B12: 0.6 mg.

**Table 2:** Sequences of the primers used for reverse transcriptase PCR analyses.

Genes	Primers (5′-3′)	ATc (°C)	Size, bp
*Occludin*	F:CTACTCGTCCAACGGGAAAG	65	150
	R:ACGCCTCCAAGTTACCACTG		
*Claudin-1*	F:GCCACAGCAAGGTATGGTAAC	65	102
	R:AGTAGGGCACCTCCCAGAAG		
*ZO-1*	F:CAGCCCCCGTACATGGAGA	65	118
	R:GCGCAGACGGTGTTCATAGTT		
*β-actin*	F:ACAATGAGCTTCGTGTTGCC	65	114
	R:CATCTCCAGAGTCCAGCACA		

ATc annealing temperature; ZO-1, zonula occludens-1.

**Table 3:** Effects of different iron sources on 35-day growth performance and diarrhea incidence in weaned piglets.

Item	FeSO_4_	FAC	Fe-Gly	*p* ^1^	*p* ^2^	*p* ^3^
BW, kg						
Day 1	6.98 ± 0.25	7.14 ± 0.22	7.14 ± 0.21	0.999	0.999	0.999
Day 21	11.75 ± 0.58	11.57 ± 0.38	12.19 ± 0.438	0.963	0.813	0.670
Day 35	18.12 ± 0.90 ^a^	18.83 ± 0.52 ^ab^	20.49 ± 0.39 ^b^	0.719	0.049	0.197
Day 1–21						
ADG, g	227.22 ± 20.44	220.70 ± 7.51	251.80 ± 21.13	0.946	0.453	0.297
ADFI, g	370.25 ± 32.31	355.83 ± 18.73	379.94 ± 51.06	0.909	0.958	0.771
F/G	1.63 ± 0.06	1.61 ± 0.07	1.52 ± 0.08	0.976	0.502	0.627
Diarrhea incidence ^1^	24.98 ± 2.35 ^a^	18.90 ± 1.31 ^ab^	14.76 ± 1.78 ^b^	0.086	0.004	0.289
Diarrhea index ^2^	0.48 ± 0.084	0.42 ± 0.035	0.36 ± 0.034	0.750	0.332	0.744
Day 21–35						
ADG, g	471.73 ± 28.98 ^a^	503.99 ± 20.14 ^a^	585.89 ± 11.99 ^b^	0.552	0.005	0.041
ADFI, g	869.88 ± 17.58 ^a^	912.44 ± 31.00 ^ab^	1001.91 ±32.87 ^b^	0.519	0.009	0.079
F/G	1.87 ± 0.09	1.81 ± 0.06	1.71 ± 0.07	0.878	0.371	0.648
Day 1–35						
ADG, g	318.38 ± 20.31 ^a^	338.87 ± 10.61 ^ab^	386.37 ± 9.14 ^b^	0.585	0.012	0.082
ADFI, g	570.12 ± 23.04	578.31 ± 9.42	628.68 ± 25.10	0.957	0.154	0.241
F/G	1.81 ± 0.05	1.71 ± 0.03	1.63 ± 0.06	0.483	0.106	0.583

*p*^1^ denotes the *p*-value for the comparison between the FeSO_4_ group and the FAC group; *p*^2^ denotes the *p*-value for the comparison between the FeSO_4_ group and the Fe-Gly group; *p*^3^ denotes the *p* value for the comparison between the FAC group and the Fe-Gly group. ^1^ Diarrhea incidence, %; ^2^ Diarrhea index, Unitless. Calculation formula detailed in the text. Diarrhea incidence and Diarrhea index were calculated only for days 1–14. Mean values followed by different letters within a row are significantly different (*p* < 0.05).

## Data Availability

The data underlying this article will be shared on reasonable request to the corresponding author.

## References

[1] Galaris D., Barbouti A., Pantopoulos K. (2019). Iron homeostasis and oxidative stress: An intimate relationship. Biochim. Biophys. Acta Mol. Cell Res..

[2] Ayuso M., Irwin R., Walsh C., Cruchten S., Ginneken C. (2021). Low birth weight female piglets show altered intestinal development, gene expression, and epigenetic changes at key developmental loci. FASEB J..

[3] Ding H., Yu X., Feng J. (2020). Iron homeostasis disorder in piglet intestine. Metallomics.

[4] Lee S.H., Shinde P., Choi J., Park M., Ohh S., Kwon I.K., Pak S.I., Chae B.J. (2008). Effects of dietary iron levels on growth performance, hematological status, liver mineral concentration, fecal microflora, and diarrhea incidence in weanling pigs. Biol. Trace Elem. Res..

[5] Huang R.Q., Yang X.J., Xie G.M., Li J., Jian Y.H., Yang J., Zhu Y.W. (2023). Effects of dietary iron sources on growth performance, iron status, Fe-containing enzyme activity and gene expression related to iron homeostasis in tissues of weaned pigs. Front. Vet. Sci..

[6] Jiang S., Dong W., Zhang Z., Xu J., Li H., Zhang J., Dai L., Wang S. (2022). A new iron supplement: The chelate of pig skin collagen peptide and Fe^2+^ can treat iron-deficiency anemia by modulating intestinal flora. Front. Nutr..

[7] Liu Y., Wu A., Yu B., He J., Yu J., Mao X., Zheng P., Luo Y., Luo J., Pu J. (2024). The influence of iron nutrition on the development of intestine and immune cell divergency in neonatal pigs. J. Anim. Sci. Biotechnol..

[8] Zhao F., Li D., Chen H., Zeng X., Lin L., Yuan H., Shan R., Chen Y. (2025). Pyrolysis of pig waste from intensive farming operations: Kinetics, product distribution, and transformation of endogenous heavy metals. J. Hazard. Mater..

[9] Brady P.S., Ku P.K., Ullrey D.E., Miller E.R. (1978). Evaluation of an amino acid-iron chelate hematinic for the baby pig. J. Anim. Sci..

[10] Guo H. (2005). Effects of Combined Use of Compound Amino Acid Chelated Iron and Inorganic Iron on Growth Performance and Blood Physicochemical Indices of Weaned Piglets. Master’s Thesis.

[11] Valcárcel A.M.C., Graciá C.M., Miró S.M., Sánchez J.M., Bermúdez C.A.G., Asensi G.D., Nicolás R.L., Pascual M.S. (2019). Iron bioavailability of four iron sources used to fortify infant cereals, using anemic weaning pigs as a model. Eur. J. Nutr..

[12] Zhuo Z. (2017). Effects of Different Iron Sources on Systemic Iron Metabolism and Their Absorption Mechanisms in the Intestine. Doctoral Dissertation.

[13] Wang J. (2020). Effects of Different Iron Sources on Iron Metabolism, Intestinal Mucosal Immunity and Ileal Microbiota Structure of Suckling Piglets. Master’s Thesis.

[14] Ma J., Liu S., Piao X., Wang C., Wang J., Lin Y.S., Hsu T.P., Liu L. (2022). Dietary Supplementation of Ferrous Glycine Chelate Improves Growth Performance of Piglets by Enhancing Serum Immune Antioxidant Properties, Modulating Microbial Structure and Its Metabolic Function in the Early Stage. Front. Vet. Sci..

[15] Dai F.W., Liu H., Huang X., Ma J., Yi Z.J. (2022). Research Progress on Iron Nutrition Regulation in Suckling and Weaned Piglets. China Feed.

[16] Cai Z., Jin S., Gong J., Diarra M., Nyachoti C.M., Jendza J., Yang C. (2024). 500 Effect of different iron sources on growth performance, gut health, and microbiota in nursery pigs. J. Anim. Sci..

[17] Shi B.Z., Fan J.J., Gu D.C., Wang D., Liu Y.L., Kang P. (2023). Effects of Different Iron Additives on Serum Biochemical Parameters and Organ Indices in Weaned Piglets. Chin. J. Anim. Sci..

[18] NRC (National Research Council) (2012). Nutrient Requirements of Swine.

[19] Che L., Hu L., Zhou Q., Peng X., Liu Y., Luo Y., Fang Z., Lin Y., Xu S., Feng B. (2020). Microbial insight into dietary protein source affects intestinal function of pigs with intrauterine growth retardation. Eur. J. Nutr..

[20] Douglas G.M., Maffei V.J., Zaneveld J.R., Yurgel S.N., Brown J.R., Taylor C.M., Huttenhower C., Langille M.G.I. (2020). PICRUSt2 for prediction of metagenome functions. Nat. Biotechnol..

[21] Langille M.G.I., Zaneveld J., Caporaso J.G., McDonald D., Knights D., Reyes J.A., Clemente J.C., Burkepile D.E., Vega Thurber R.L., Knight R. (2013). Predictive functional profiling of microbial communities using 16S rRNA marker gene sequences. Nat. Biotechnol..

[22] Song Z.H., Xiao K., Ke Y.L., Jiao L.F., Hu C.H. (2015). Zinc oxide influences mitogen-activated protein kinase and TGF-β1 signaling pathways, and enhances intestinal barrier integrity in weaned pigs. Innate Immun..

[23] Chen F.Q., Ji F., Cheng M.J., Tang H.O., Meng X.L. (2008). Effects of Different Iron Sources on Growth Performance, Immune Function and Iron Nutritional Status of Weaned Piglets. China Anim. Husb. Vet. Med..

[24] Zhang B., Li H. (2000). Effects of Different Iron Sources on Growth, Metabolism and Environment of Suckling Piglets. Chin. J. Appl. Ecol..

[25] Gao Q., Zhang Y., Wu Y., Gu D., Chen J., Yin C., Wu H., Zhu D., Chen D., Wu A. (2025). Dietary Fe-Gly supplementation attenuates enterotoxigenic Escherichia coli (ETEC)-induced inflammation response and intestinal barrier dysfunction in piglets. Front. Vet. Sci..

[26] Huang L., Hu C., Sun J., Lyu L., Zhang L., Li X., Zhang X., Liao X. (2023). Effects of Bioactive Selenium on Growth Performance, Tissue Selenium Content, Antioxidant Capacity and Meat Quality of Finishing Pigs of Different Breeds. Chin. J. Anim. Nutr..

[27] Lin D., Zhang Y., Xiong Q., Zhang L., Cheng S., Yu J., Ahmad M., Ni Y.L., Xu S., Luo H. (2025). Improvement of stability and antioxidant capacity of peptide—Iron complexes by sonication. Food Chem..

[28] Yan X.H., Xie Y., Liu H.B., Huang M., Yang Z., An D.Q., Jiang G.J. (2023). Iron accumulation and lipid peroxidation: Implication of ferroptosis in diabetic cardiomyopathy. Diabetol. Metab. Syndr..

[29] Jiang Y.W., Xiao L.B., Fu W.W., Tang Y.X., Lertnimitphun P., Kim N., Zheng C.W., Tan H.S., Lu Y., Xu H.X. (2020). Gaudichaudione H Inhibits Inflammatory Responses in Macrophages and Dextran Sodium Sulfate-Induced Colitis in Mice. Front. Pharmacol..

[30] Wu A., Feng B., Yu J., Yan L., Che L., Zhuo Y., Luo Y., Yu B., Wu D., Chen D. (2021). Fibroblast growth factor 21 attenuates iron overload-induced liver injury and fibrosis by inhibiting ferroptosis. Redox Biol..

[31] Kitazawa M., Iwasaki K. (1999). Reduction of ultraviolet light-induced oxidative stress by amino acid-based iron chelators. Biochim. Biophys. Acta Gen. Subj..

[32] Inoue K., Sakano N., Ogino K., Sato Y., Wang D.H., Kubo M., Takahashi H., Kanbara S., Miyatake N. (2013). Relationship between ceruloplasmin and oxidative biomarkers including ferritin among healthy Japanese. J. Clin. Biochem. Nutr..

[33] Parra-Flores P., Riquelme J.A., Valenzuela-Bustamante P., Leiva-Navarrete S., Vivar R., Cayupi-Vivanco J., Castro E., Espinoza-Pérez C., Ruz-Cortés F., Pedrozo Z. (2019). The Association of Ascorbic Acid, Deferoxamine and N-Acetylcysteine Improves Cardiac Fibroblast Viability and Cellular Function Associated with Tissue Repair Damaged by Simulated Ischemia/Reperfusion. Antioxidants.

[34] Li J., Yu C., Wang P., Li Y., Xu C., Zhang C., Pan H., An Q. (2025). Effects of Dietary Iron Content on Iron Deposition in Internal Organs and Intestinal Barrier Function in Wujin Pigs. Feed Res..

[35] Uddin M.K., Mahmud M.R., Hasan S., Peltoniemi O., Oliviero C. (2023). Dietary micro-fibrillated cellulose improves growth, reduces diarrhea, modulates gut microbiota, and increases butyrate production in post-weaning piglets. Sci. Rep..

[36] Gu K., Wu A., Liu C., Yu B., He J., Lai X., Chen J., Luo Y., Yan H., Zheng P. (2025). Absence of gut microbiota alleviates iron overload-induced colitis by modulating ferroptosis in mice. J. Adv. Res..

[37] McMillen S., Thomas S., Liang E., Nonnecke E.B., Slupsky C., Lönnerdal B. (2022). Gut Microbiome Alterations following Postnatal Iron Supplementation Depend on Iron Form and Persist into Adulthood. Nutrients.

[38] Marzet C.B., Burgos F., Del Compare M., Gerold I., Tabacco O., Vinderola G. (2022). Approach to probiotics in pediatrics: The role of Lactobacillus rhamnosus GG. Arch. Argent. Pediatr..

[39] Cai J., Tang Z., Deng H., Sun Z., Lai X. (2014). Research Progress on the Mechanism of Antimicrobial Peptides in Animal Intestinal Mucosa Maintaining Microbiota Balance. Chin. J. Anim. Nutr..

[40] Das N.K., Schwartz A.J., Barthel G., Inohara N., Liu Q., Sankar A., Hill D.R., Ma X.Y., Lamberg O., Schnizlein M.K. (2020). Microbial Metabolite Signaling Is Required for Systemic Iron Homeostasis. Cell Metab..

[41] Li W.L., Xin Y., Zhao L.J. (2008). Induction of IL-1β, IL-6, and TNF-α Production in Fibroblast L929 Cells by Fimbriae of *Actinomyces naeslundii* Strain ATCC 19246. J. China Med. Univ..

